# Supplementary material for: Circadian Gene PER2 Silencing Downregulates PPARG and SREBF1 and Suppresses Lipid Synthesis in Bovine Mammary Epithelial Cells
Source: Biology (Basel). 2021 Nov 24;10(12):1226. doi: 10.3390/biology10121226 (PMC8698707; doi:10.3390/biology10121226)
Supplement: Supplementary file 1 [file biology-10-01226-s001.zip › biology-1416563-supplementary.pdf]

Table S1. The sequences of siRNAs for PER2 silencing

siRNA	sense	antisense
siRNA-PER2-a	CCAGAAACCUUUGGCCUAATT	UUAGGCCAAAGGUUUCUGGTT
siRNA-PER2-b	GCAACCCUGCUUCAGAAAUTT	AUUUCUGAAGCAGGGUUGCTT
siRNA-PER2-c	GGAGGAGACCGAAACUUGUAATT	UUACAGUUUCGGCUCUCCUCCTT
Negative control	UUCUCCGAACGUGUCACGUTT	ACGUGACACGUUCGGAGAATT

Table S2. The Primer sequences of apoptosis pathway genes

Gene	NCBI accession	Primer sequence (F:5'->3')	Primer sequence (R:5'->3')
PER2	NM_001192317.1	agcgtgttccatagctccac	atctcgctctcgtggcttt
Cell apoptosis-related genes			
Caspase 3	NM_001077840.1	ggagacgggttgaggacaat	cagagtgcccaactgactga
Caspase 8	NM_001045970.2	catcatctatggctccgatg	gagtcggtctcaacggctac
P53	NM-174201.2	atttacgcgcggagtatttg	ccagtgtgatgatggtgagg
β-actin	U02295.1	actgttagctgcgttacaccctt	tgctgtcaccttcaccgttcc
Lipid synthesis-related genes			
SCD	NM_173959.4	taccacgttcttcattgattgc	acgtcatctttagcatcctggt
LPL	NM_001075120.1	gaccccctggtgaatgtgtgtg	gccatatgagaacatgccgca
ACACA	NM_174224.2	ttaatcaacactccgtatgtgaccaa	gactgccgaaacatttctgggat
SREBF1	NM_001113302.1	acgccatcgagaaacgctac	gtgcgcagactcaggttctc
mTOR	XM_002694043.6	atgctgtccctggtccttatg	gggtcagagagtggccttcaa
FAS	U34794.1	gaacatgggctagaagtggaac	ctattgggatgaggaggatcag
PPARG	NM_181024.2	taccaaagtgcaatcaaagtgg	atagtggaaccctgacgcttta
LPIN1	NM_001206156.2	ttcccgaccgtcaacaccta	caacgggctggactctttca
β-actin	U02295.1	actgttagctgcgttacaccctt	tgctgtcaccttcaccgttcc
